# Detection of *Plasmodium falciparum* in laboratory-reared and naturally infected wild mosquitoes using near-infrared spectroscopy

**DOI:** 10.1038/s41598-021-89715-1

**Published:** 2021-05-13

**Authors:** Dari F. Da, Ruth McCabe, Bernard M. Somé, Pedro M. Esperança, Katarzyna A. Sala, Josua Blight, Andrew M. Blagborough, Floyd Dowell, Serge R. Yerbanga, Thierry Lefèvre, Karine Mouline, Roch K. Dabiré, Thomas S. Churcher

**Affiliations:** 1 Institut de Recherche en Sciences de la Santé, Direction Régionale, 399 avenue de la liberté, 01 BP 545 Bobo-Dioulasso 01, Burkina Faso; 2 MRC Centre for Global Infectious Disease Analysis, Infectious Disease Epidemiology, Imperial College London, London, W2 1PG UK; 3 Division of Microbiology and Parasitology, Department of Pathology, Cambridge University, Cambridge, CB2 1QP UK; 4 Department of Life Sciences, Imperial College London, Sir Alexander Fleming Building, Exhibition Road, South Kensington, London, UK; 5 Stored Product Insect and Engineering Research Unit, United States Department of Agriculture/Agricultural Research Services, Center for Grain and Animal Health Research, Manhattan, KS USA; 6 MIVEGEC, Montpellier University, IRD, CNRS, Montpellier, France; 7 Laboratoire Mixte International Sur Les Vecteurs (LAMIVECT), Bobo Dioulasso, Burkina Faso; 8 Centre de Recherche en Écologie et Évolution de la Santé (CREES), Montpellier, France

**Keywords:** Biological techniques, Biotechnology, Computational biology and bioinformatics, Molecular biology, Medical research, Mathematics and computing

## Abstract

There is an urgent need for high throughput, affordable methods of detecting pathogens inside insect vectors to facilitate surveillance. Near-infrared spectroscopy (NIRS) has shown promise to detect arbovirus and malaria in the laboratory but has not been evaluated in field conditions. Here we investigate the ability of NIRS to identify *Plasmodium falciparum* in *Anopheles coluzzii* mosquitoes. NIRS models trained on laboratory-reared mosquitoes infected with wild malaria parasites can detect the parasite in comparable mosquitoes with moderate accuracy though fails to detect oocysts or sporozoites in naturally infected field caught mosquitoes. Models trained on field mosquitoes were unable to predict the infection status of other field mosquitoes. Restricting analyses to mosquitoes of uninfectious and highly-infectious status did improve predictions suggesting sensitivity and specificity may be better in mosquitoes with higher numbers of parasites. Detection of infection appears restricted to homogenous groups of mosquitoes diminishing NIRS utility for detecting malaria within mosquitoes.

## Introduction

Mosquito-borne diseases continue to cause widespread suffering world-wide. Malaria cases are thought to have risen in the last few years following two decades of decline^1^ whilst the public health impact of arboviruses such as dengue, chikungunya and zika continues to increase^2^. Killing the mosquito vector is the most effective current method for controlling these diseases^3^ and it is important to monitor infection in local mosquito populations to understand the efficacy of control interventions, track disease trends and provide warnings of outbreaks.

Entomological monitoring is costly and time consuming. The short life-expectancy of mosquitoes means that typically fewer than 5% of vectors are infectious even in highly endemic regions^4^. This means that high number of insects need to be tested to generate reliable estimates. Unfortunately, there are no cheap and easy-to-use methods of detecting pathogens in mosquitoes. In malaria, the presence of infectious sporozoites is determined either by manual salivary gland dissection using a microscope or through molecular methods such as PCR (polymerase chain reaction) or ELISA (enzyme-linked immunosorbent assay)^5–7^. All these techniques are laborious and are therefore costly for large sample size whilst PCR also requires well-equipped laboratories and expensive reagents.

Near-infrared spectroscopy (NIRS) is a fast, non-destructive and reagent-free scanning technique which has been shown to detect mosquitoes infected with rodent models of malaria^8^, laboratory strains of human malaria^9^, dengue, zika^10^and the endosymbiont Wolbachia bacteria^11^. Mosquitoes are scanned at different wavelengths in the near-infrared region of the electromagnetic spectrum and a chemometric model is used to convert spectra into estimates of pathogen prevalence. All previous NIRS infection works have been conducted on laboratory reared mosquitoes of similar age and using laboratory strains of pathogen. The accuracy of these diagnostics has been evaluated on a sub-set of the same group of mosquitoes, which is likely to overestimate sensitivity and specificity. There is also evidence that the technique may lose accuracy when there is more diverse field derived parasites and mosquitoes^12^. Here we evaluate the ability of NIRS to determine *Anopheles coluzzii* infection status with wild *Plasmodium falciparum* isolates circulating in Burkina Faso. This is initially conducted with laboratory-reared *Anopheles* before evaluating the ability of the models to detect the parasite in wild caught mosquitoes infected naturally in the field. It is unclear whether NIRS is detecting the presence of parasite biomass or a physiological change in the mosquito. Here we devise a comprehensive set of experiments which would enable the differentiation of mosquitoes which (1) have fed on malaria infected blood, (2) are infected with oocyst life-stages (visible in Burkina Faso from 3 to 11 days from infection)^13^ and (3) are infectious with salivary gland sporozoites. Sporozoites are the most epidemiologically important parasite life stage although evaluation of control interventions might be easier with earlier life-stages which have a higher prevalence in wild mosquito populations and therefore require lower number of mosquitoes to generate sufficiently precise estimates.

## Results

### Laboratory-reared mosquitoes

**Figure 1:**
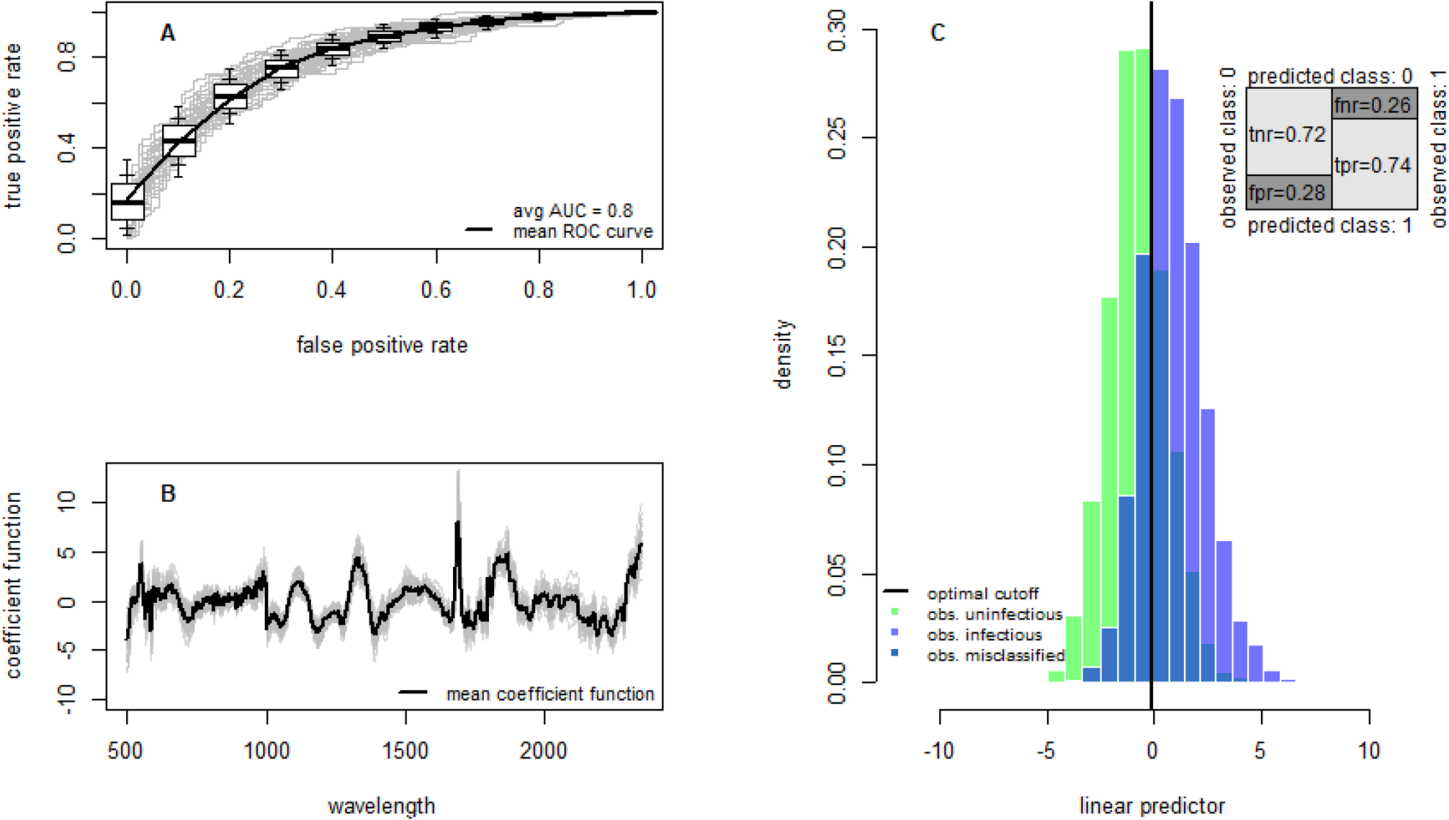
The ability of NIRS to predict laboratory-reared mosquitoes infectious with wild parasites. All models were trained on sporozoite positive and sporozoite negative laboratory reared mosquitoes using all the data presented in Table 1. (**A**) Receiver operating characteristic (ROC) curve illustrating the diagnostic ability of the best-fit model. Overall performance is given by the average area under the ROC curve (AUC). Figure illustrates the false positive and true positive rates achievable for different classification probability thresholds. A theoretical perfect diagnostic would be in the top left corner. Average ROC curve shown by the solid line with boxplots showing the variability for 50 randomizations of the training, validation and testing datasets (horizontal black line shows the median whilst the 25th/75th, 15th/85th and 5th/95th percentiles are shown by box edges, inner and outer whiskers, respectively). (**B**) Coefficient functions for the best fit model for each of the 50 dataset randomizations (grey lines) and the overall average (black line). (**C**) Histogram showing the predicted status of tested mosquitoes that were infectious (light blue colored bars) or uninfectious (green bars).Vertical solid black line indicates the best threshold for differentiating between infectious or uninfectious mosquitoes. Darker blue bars indicates where the two distributions overlap and show those mosquitoes misclassified—false negatives are shown to the left of the optimal classification threshold line and false positives to the right. Inset shows the confusion matrix illustrating the different error rates: true negative rate (tnr, specificity); false negative rate (fnr); false positive rate (fpr); and true positive rate (tpr, sensitivity).

NIRS can identify mosquitoes infected and infectious with wild malaria parasites with relatively high accuracy. A total of 2452 *An. coluzzii* mosquitoes of ages ranging from 3 to 27 days were used to train the model (Table 1). Overall within-sample accuracy, defined as the percentage of mosquitoes correctly classified, for detecting sporozoite positive mosquitoes (uninfectious vs infectious) was 73% (sensitivity = 74%, specificity = 72%, Fig. 1, Table 2). NIRS was also able to differentiate between uninfected mosquitoes and those with either oocysts or sporozoites (uninfected vs infected), though with slightly lower accuracy (accuracy = 71%, sensitivity = 71%, specificity = 70%, Fig. S1). Accuracy was similar for mosquitoes infected on Day 3 or Day 6 after emergence (accuracy = 74% and accuracy = 72%, respectively, for uninfectious vs infectious; accuracy = 73% and accuracy = 72%, respectively, for uninfected vs infected).

**Table 1:** The number of laboratory and field mosquitoes analyzed.

	Days since feeding (laboratory-reared mosquitoes)											Wild caught mosquitoes		
	3	5	7	9	11	13	15	17	19	21	Total	Longo	Klesso	Total
**Unexposed to infectious gametocytes**														
Inactivated blood	100	100	140	100	100	100	109	90	45	20	904	NA	NA	NA
**Fed infectious gametocytes**														
Uninfected	147	110	106	75	73	70	58	40	39	1	719	2445^a^	80	2525
Infected (oocysts)	45	88	91	112	104	102	101	90	66	30	829	387	25	412
Infectious^b^ (sporozoites)	0	0	0	51	92	102	101	90	66	30	532	302	21	323
Total	292	298	337	287	277	272	268	220	220	51	2452	2832	105	2937

All data were *Anopheles coluzzii* mosquitoes infected with wild strains of *Plasmodium falciparum.*

^a^Blood source unknown as mosquitoes were collected potentially exposed.

^b^All infectious mosquitoes were classified as also infected (whether or not oocysts were visible).

**Table 2:** Summary of overall accuracy of the different NIRS models for predicting presence of sporozoites.

Model trained on	Within-sample accuracy				Model predicting	Out-of-sample accuracy						
	Best model (Q)	Accuracy (std error)	TPR	TNR		Best within-sample model			Best out-of-sample model			
						Accuracy (std error)	TPR	TNR	Best model (Q)	Accuracy (std error)	TPR	FPR
Laboratory mosquitoes	GLM (11)	73% (0.02)	74%	72%	Field mosquitoes (all)	50% (0.01)	56%	44%	fsGLM (4)	52% (0.007)	52%	51%
Field mosquitoes (all)	fsGLM (2)	51% (0.04)	65%	37%		NA	NA	NA	NA	NA	NA	NA
Field mosquitoes (V1)	pGLM (2)	51% (0.04)	57%	46%	Field mosquitoes (V2)	51% (0.05)	58%	45%	fpGLM (2)	52% (0.05)	60%	43%
Field mosquitoes (V2)	fpGLM (5)	47% (0.1)	28%	64%	Field mosquitoes (V1)	51% (0.02)	30%	71%	fspGLM (5)	51% (0.02)	39%	63%

Models were trained on either laboratory or field mosquitoes, either all mosquitoes grouped together (all) or separately for mosquitoes from the villages of Longo (V1) or Klesso (V2). The number of PLS components (*Q*) is presented alongside overall models accuracy (the percentage of mosquitoes correctly classified), the true positive rate (TPR) and false positive rate (FPR). This is shown for either within sample accuracy (where the same group of mosquitoes were used to train/validate and test the model) or out-of-sample accuracy (where a different group of wild caught mosquitoes were used). For within-sample accuracy different individual mosquitoes were used to train, validate and test the model though out-of-sample evaluation provides a more robust test as different groups (i.e. laboratory vs field or different field locations) were used to assess accuracy. Two different models are presented for out-of-sample accuracy, either the most accurate either within-sample or out-of-sample (which tend to be more generalizable and have lower numbers of components, denoted *Q*).

It is unclear whether NIRS is detecting parasite biomass or some metabolic or physiological response of the mosquito. NIRS had relatively poor accuracy differentiating between uninfected/uninfectious mosquitoes fed on infectious and heat inactivated blood (balancing for mosquito age between groups, accuracy = 64%). This suggests the presence of infectious gametocytes is not initiating an immunological response subsequently detected by NIRS or that NIRS is directly detecting developing alive parasite.

### Wild mosquitoes using models trained on laboratory mosquitoes

Models trained on laboratory-reared mosquitoes infected with wild parasites were unable to predict infection status of wild caught mosquitoes. Overall out-of-sample accuracy for detecting wild caught sporozoite positive mosquitoes (uninfectious vs infectious) using the model with best within-sample accuracy was accuracy = 50% (sensitivity = 56%, specificity = 44%). Varying the machine learning method to reduce overfitting improves accuracy (Table 2) though predictions are still very poor (accuracy = 52%, sensitivity = 52%, specificity = 51%).

### Wild mosquitoes using models trained on wild mosquitoes

To determine whether there was any difference in the spectra from infectious and uninfectious mosquitoes models were trained on wild-caught *An. coluzzii* mosquitoes alone. NIRS was unable to differentiate infectious or infected-infectious mosquitoes with any accuracy (accuracy of 51% and 51%, respectively). Examining mosquitoes from the same village did not substantially improve within- or out-of-sample predictions (Table 2).

### Impact of mosquito age on detection

NIRS can differentiate the age of laboratory-reared mosquitoes with high accuracy^14,15^. Previous laboratory studies investigating infection have only used mosquitoes of the same age (3–6 days post emergence). All mosquitoes were then same aged mosquitoes (3–6 days post emergence). Then, all fed mosquitoes were dissected at the same time point: between 6 and 9 days for oocysts counting or from 10 days for sporozoites detection^17^. To generate more robust results a range of different aged mosquitoes were compared here which could in part account for somewhat lower accuracy than previous studies. Field mosquitoes will have to be greater than 13 days old if an extrinsic incubation period of 10 days is assumed. The inability of NIRS to detect wild infectious mosquitoes could be associated with the informative region of the spectra interacting with wavelengths that change with mosquito age. To test this hypothesis models were trained on laboratory-reared mosquitoes using a two-step process. Firstly, it was determined whether an individual mosquito was > 13 days old which the model achieved with high accuracy (within-sample accuracy = 84%). Secondly, older mosquitoes were then used to train the model for infectiousness which was again achieved with high accuracy (within-sample accuracy = 76%). Nevertheless, repeating the two-step process on field mosquitoes failed to improve model predictions as it failed to identify infectious mosquitoes in those previously defined as > 13 days old. This would suggest that the different age distributions of mosquitoes in the calibration and test data sets cannot explain the contrasting results of the laboratory and field data and that age is not confounding our result.

### Impact of parasite intensity on diagnosis

**Figure 2:**
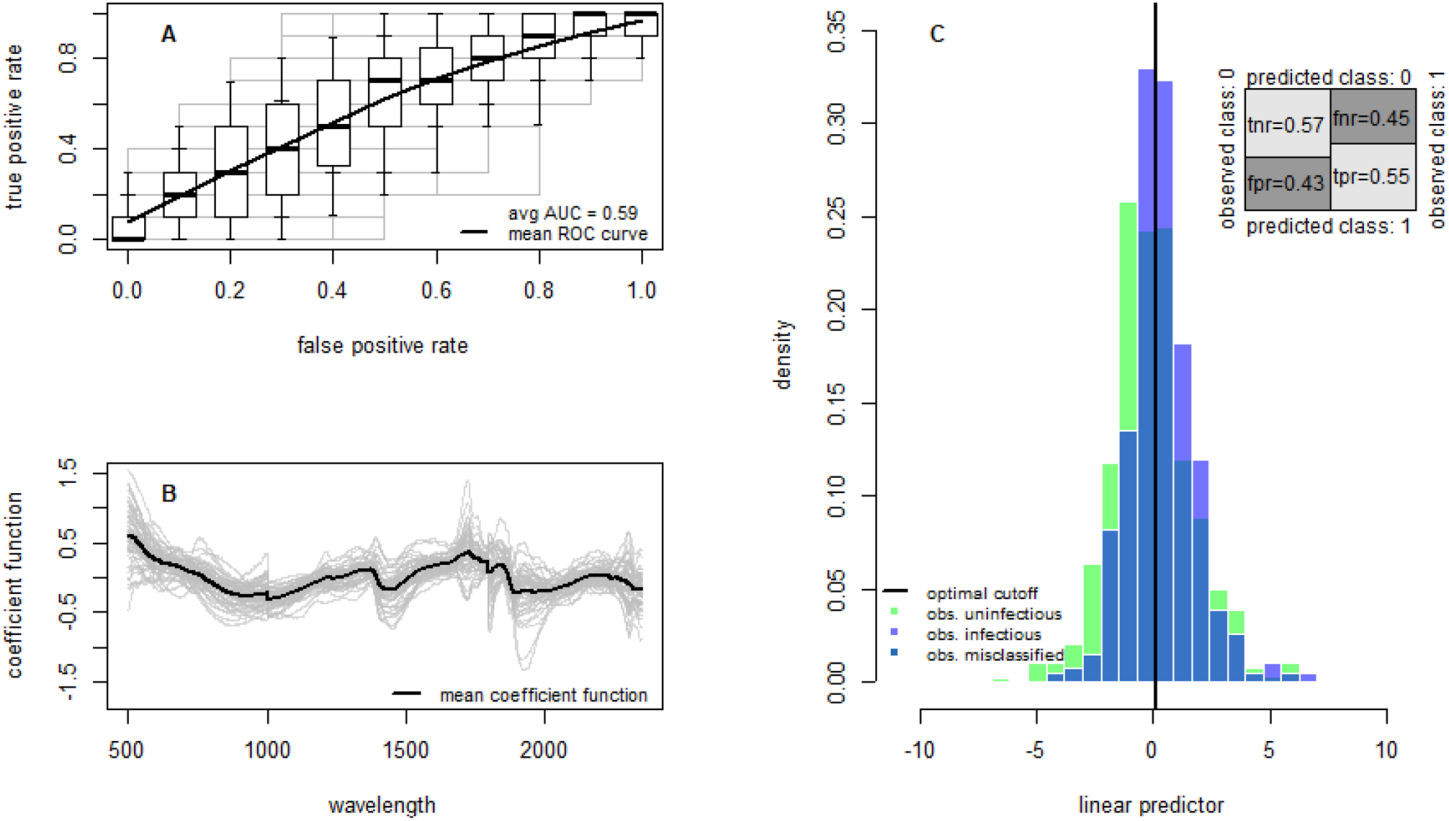
The ability of NIRS to predict field caught mosquitoes with high number of sporozoites. All models were trained using mosquitoes infected in the wild and were either sporozoite positive mosquitoes with > 20 sporozoite per *Anopheles* (20 gene copy number as defined by qPCR) or sporozoite negative mosquitoes (Table 1). (**A**) The receiver operating characteristic (ROC) curve for the best-fit model demonstrating how the false positive and true positive rates vary for different for different classification probability thresholds. Overall performance is given by the average area under the ROC curve (AUC). A perfect model with 100% sensitivity and specificity would be in the top left corner. Solid line shows the average ROC curve with boxplots showing the variability for 50 randomizations of the training, validation and testing datasets (with box edges, inner and outer whiskers showing 25th/75th, 15th/85th and 5th/95th percentiles, respectively; and the black line inside the box showing the median/50th-percentile). (**B**) Coefficient functions for the best fit model for each of the 50 dataset randomizations (grey lines) and the corresponding average (black line). (**C**) The histogram of the estimated linear predictor for the test mosquitoes, the green and light blue colored bars indicate the true class, showing the model’s ability to separate the two groups of mosquitoes. Vertical black line indicates the best threshold for differentiating infectious or uninfectious mosquitoes. The darker blue shaded area where the two distributions overlap corresponds to mosquitoes which have been misclassified—false negatives to the left and false positives to the right of the optimal classification threshold. Inset shows the confusion matrix reporting the different error rates: tnr, true negative rate (specificity); fnr, false negative rate; fpr, false positive rate; and tpr, true positive rate (sensitivity).

The number of sporozoites in wild caught mosquitoes may be substantially lower than those infected through a direct membrane feeding assay. Quantitative PCR investigating sporozoite intensity was only conducted on wild caught mosquitoes. Nevertheless, the mean number of oocysts per oocyst-positive mosquito was 8.39 in the laboratory experiments and 3.05 recorded from wild caught mosquitoes. This difference in the intensity of infection may cause the spectra from laboratory and field-reared to differ. To investigate this the models trained on field mosquitoes were rerun comparing uninfectious mosquitoes with those infectious mosquitoes with > 20 sporozoites per mosquito (as determined by quantitative PCR). Accuracy of the models does improve suggesting parasite intensity might be a contributing factor, though the predictive ability is still poor (accuracy = 56%, sensitivity = 55%, specificity = 57%, Fig. 2), and the number of naturally-infected mosquitoes with high sporozoite loads available to fit the model was relatively low (37 *An. coluzzii*). Similarly improved results were seen when models trained on laboratory mosquitoes were used to predict wild caught mosquitoes which were either uninfectious or highly infectious (accuracy = 64%, sensitivity = 67%, specificity = 61%, Fig. S2).

## Discussion

We show that NIRS can detect the presence of natural malaria parasites in laboratory-reared mosquitoes but cannot differentiate between uninfectious or infectious wild mosquitoes with any accuracy. Accuracy of predicting the infection status of wild mosquitoes remains poor regardless of whether mosquitoesmodels were trained on either laboratory-reared or wild mosquitoes. The failure of NIRS to differentiate between infectious and uninfectious wild mosquitoes suggests the technique is unlikely to be able to detect sporozoites in the field. A large number of mosquitoes (nearly 3000 in total, with over 300 mosquitoes sporozoite positive) and a variety chemometric approaches^16^ were used in the analysis. Though it cannot be discounted that larger sample sizes and different machine learning methods^18,19^ may improve predictions, the lack of differentiation between groups suggests substantial improvements are unlikely.

The reason why NIRS was better able to predict infection in laboratory-reared mosquitoes than wild field-infected specimens remains unclear. In the field, sporozoite positive mosquitoes are likely to be older than the average age in the mosquito population due to the relatively long extrinsic incubation period of the parasite. Previous laboratory studies have only compared infectious and uninfectious mosquitoes of the same ages so regions of the spectrum informative for age could also be indicative of infection status. Using the model to predict mosquito age first and then evaluating infectious status in the older mosquitoes did not improve identification of sporozoite positive wild mosquitoes. This would suggest that differences in the age distribution may not be the cause of the discrepancy though the ability of NIRS to evaluate the age of wild caught mosquitoes has not currently been evaluated in the field. Indeed, the inability of NIRS to detect sporozoites, which will on average be in older mosquitoes, questions the ability of the technique to determine age in field populations, though further investigation is necessary. Beside age heterogeneity, other factors including larval breeding site diversity, blood-meal and sugar sources, physiological and nutritional status may explain why NIRS is poorly able to determine the infection status of wild mosquitoes.

Results show that direct membrane feeding experiments on average generate substantially higher intensity infections than that observed in wild mosquitoes. This difference in the quantity of parasite biomass could be a factor contributing to the lower accuracy in field mosquitoes. This hypothesis is supported by the accuracy improving when comparing uninfectious vs highly infectious wild infected mosquitoes, though only 37 mosquitoes were identified with more than 20 sporozoites in the salivary gland were identified by qPCR so further improvements might be seen with larger sample sizes. It is unclear whether in the laboratory, NIRS is detecting parasite biomass or an immunological response to the parasite. The lack of differences in the spectra of uninfected mosquitoes fed infectious blood or heat inactivated blood to kill gametocytes suggests that this life-stage is not initiating the immunological reaction, though it cannot be discounted that infertile gametocytes could still initiate the response. Here we were able to identify infected (positive for oocysts and sporozoites) and infectious (positive for sporozoites) with similar accuracy. This is consistent with previous work using laboratory strains of the same parasite which was able to identify the presence of both oocysts and sporozoites^9^.

Promising laboratory results and the ease and utility of the technique means that NIRS could substantially improve monitoring of mosquito populations in the wild. The technique has the potential to determine mosquito species and age at the same time though unfortunately the evidence presented here suggests that it cannot detect whether a mosquito contains the malaria parasite as well. The need to examine large numbers of mosquitoes and the high cost of molecular methods means that there may be some utility in triaging mosquitoes using NIRS before suspected infections are confirmed using other methods. NIRS and other spectrometry methods such as mid-infrared spectroscopy^20,21^ could still substantially revolutionize the monitoring of wild mosquito populations. Nevertheless, the work presented here joins a growing body of evidence that^12^ highlights the problems associated with transferring these potentially useful entomological tools from the laboratory to the field.

## Materials and methods

### Experimental design

A comprehensive set of laboratory experiments were designed to understand the sensitivity and specificity of NIRS to detect different life-stages of the parasite inside laboratory-reared and field mosquitoes. Previous work has shown that NIRS can detect oocysts and sporozoites 7 and 14 days post infection, respectively^9^, in laboratory strains of the parasite and mosquito. Nevertheless, it is unclear whether it is detecting parasite biomass directly or a physiological change in infected mosquitoes which could be initiated by earlier life-stages (for example the ookinete stage which penetrates the mosquito mid-gut wall). To disentangle the possible cause and understand how the likelihood of detecting the parasite changes with mosquito age and time since infection mosquitoes are fed infectious and non-infectious blood on either day 3 or 6 following emergence and scanned every other day until all mosquitoes have died. Ethical approval was gained from Imperial College Research Ethics Committee (18IC4859) and “Comité d’Ethique Institutionnel pour la Recherche en Sciences de la Santé, Burkina Faso” (clearance A018-2017/CEIRES). The protocols and associated procedures were conformed to the current international legislation and recommendations, including bioethics specificities in Burkina Faso.

### Laboratory mosquitoes

A total of 2483 *Anopheles coluzzii* females were exposed to malaria using a direct membrane feeding assays (DMFAs). Blood with *Plasmodium falciparum* gametocytes was obtained from three volunteer children (aged 5–11 years) naturally infected with malaria living in villages surrounding Bobo-Dioulasso, after obtaining their parent/guardian’s informed consent. Stratified gametocyte densities (low, medium and high gametocytemia) were required expecting to generate various infection level in experimental groups of *Anopheles*). We therefore included three volunteers with 32, 136 and 1456 gametocytes per µL in venous blood. For each experiment replicate, 8–16 mL of venous blood was drawn in heparinized tubes and immediately centrifuged at 3000 rpm for 3 min to remove the supernatant, replacing it with non-immune serum from a European AB+ donor to increase infection rates. Three and six days old female mosquitoes from an outbred *Anopheles coluzzii* local colony were starved overnight and fed on the blood mixture through pre-warmed membrane feeders for 30 min. Fully fed females were sorted and maintained in cages at 28 °C ± 2, 80% ± 05 RH with 10% glucose solution. From day 3 to 21 post-blood meal mosquitoes were killed by chloroform vapor and immediately scanned. Mosquitoes killed 3–11 days from blood-feeding were immediately dissected using a light microscope and the number of oocysts on the midgut were counted. Mosquitoes killed 9–21 days were also assessed for salivary gland sporozoites using quantitative PCR^22^. A control group of uninfected and uninfectious *Anopheles* were generated by feeding some females with gametocytes inactivated blood^23^. This was performed by heating a sample of the same blood used to infect mosquitoes at 45 °C for 20 min to kill all gametocytes to provide an uninfectious control feed.

### Wild mosquitoes

Mosquitoes were caught in the houses of two villages in the Bobo-Dioulasso region of Burkina Faso. The villages of Longo and Klesso were 120 km apart to allow the robustness of the method over space to be assessed. In addition to being easily accessibility from Bobo-Dioulasso, these two villages had mosquito and human prevalence well characterized in a previous study (Bompard et al.^13^). Wild mosquitoes were caught early in the morning by the technicians using a mouth aspirator in the living room of human dwellings^24^ and transferred to the laboratory. They were maintained in cages (30 × 30 × 30 cm) in laboratory conditions (28 °C ± 2, 80% ± 05 RH with 10% glucose solution) during 3 or 7 days periods before the next step. These days were chosen to allow mosquitoes to digest their last blood-meal (to enable dissection and enumeration of oocysts), increase the number of sporozoite positive mosquitoes and match previous work (Bompard et al.^13^). At these indicated periods, the *Anopheles* females were scanned using the spectrometer and their midgut was immediately dissected under a stereomicroscope to determine oocyst prevalence. The remaining carcass head-thorax was molecular analyzed for *Anopheles* species identification^25^ and sporozoites detection in salivary glands^22^. Only *Anopheles coluzzii* mosquitoes identified by PCR were included for statistical analysis for NIRS *P. falciparum* infection detection. All mosquitoes positive *P. falciparum* were further analysed through quantitative PCR to determine sporozoite intensity. qPCR analysis of gDNA was used to quantify the gene copy number in the mosquitoes. Analysis was performed in triplicate in 10ul reaction using BioRad SSO Advanced Universal Sybr Green Supermix (BioRad, 1725272) and the Roche LightCycler 480. Primers were designed to amplify fragment of *Plasmodium falciparum* HSP70 gene with the following sequences: forward primer 5′-GAGGTATGCCCGGTGGAATG-3′; reversed primer 5′-CTGTTGGTCCACTTCCAGCT-3′. Reactions were 40 cycles using following conditions: initial denaturation for 3 min at 95 °C, and 40 cycles of 10 s denaturation at 95 °C and 20 s amplification at 60 °C. The number of HSP70 gene copies in gDNA extracted from mosquito was calculated from their respective Ct value based on plasmid standard curve. The standard curve was generated from serial dilutions of a plasmid pGEMPfHSP70 containing *Plasmodium falciparum* HSP70 gene. Mosquitoes with over 20 gene copy numbers were classified as being highly infectious.

### Mosquito scanning

Mosquitoes were killed with chloroform vapor and scanned using a LabSpec4 Standard-Res i (standard resolution, integrated light source) near-infrared spectrometer and a bifurcated reflectance probe mounted 2 mm from a spectralon white reference panel (ASD Inc., Westborough, Massachusetts, USA). Absorbance at 2151 wavelengths from 350 to 2500 nm of the electromagnetic spectrum was recorded using RS3 spectral acquisition software (ASD Inc., Westborough, Massachusetts, USA^17^) which averaged spectra from 20 scans. All mosquitoes were scanned on both sides centering the light probe on the head and thorax region.

### Statistical analysis

Machine learning methods were used to construct binomial logistic regression models using maximum likelihood. The mean of the two spectra from each mosquito were used in the analysis. Spectra were then trimmed to values corresponding to 500–2350 nm to remove the excess noise arising from the sensitivity of the spectrometer at the ends of the near-infrared range^26^. These spectra were analysed using partial least squares regression (PLS), a statistical technique utilising the covariance between the spectra and infection status in order to extract the most informative elements within a much smaller dimension. This method generates different numbers of principal components which are linearly independent and used as the explanatory variables in the regression model. An upper limit of 20 components was enforced, with the optimal number of components being determined via ordinary cross-validation.

In conjunction with the use of PLS, three additional techniques were used to further improve model generalisability through ensuring as smooth a coefficient function as possible: functional representation of the spectra, spectral smoothing and penalised regression^16^. The utility of each technique was considered independently as well as in conjunction with one another. Representing the spectra with a set of basis functions of size *k*, written as a proportion of the total number of spectral variables, both removes excess noise from the data and increases computational efficiency by reducing the dimensionality of the data. Spectral smoothing achieves a similar effect but through the use of B-spline functions and with no reduction in dimensionality. Finally, ridge regression, a form of penalisation with a squared penalty term, shrinks the values of the coefficient function and favours models with lower numbers of explanatory variables.

The number of observations belonging to each class was often imbalanced so the training and independent testing data were sampled from to enforce the same number of observations from each class and optimise the model’s performance both within- and out-of-sample. The balanced training data was further split into training, cross-validation and testing subsets of sizes 50%, 25% and 25% respectively. This process was repeated 50 times to minimise the possibility of sampling error from both the balancing and data splitting. For each of the 50 iterations multiple sub-models were fit using the training subset, (with 2–20 components), and for those models deploying penalised regression, with ten exponentially increasing values of the penalty parameter from 0.01 to 20. The accuracy of each was tested using the cross-validation subset to determine which option maximised the area under the receiver operating characteristic curve (AUC, value closer to 1 indicating better performance). Once the maximal sub-model were identified, the sub-model with a lesser number of components with AUC value within τ of the maximised AUC value was selected as the overall optimal model that is presented in the results. By applying this finalised model to the testing subset, the critical threshold minimising the error arising from classifying these mosquitoes as infectious or uninfectious was estimated. The error structure was calculated as the number of false negative and false positive predictions divided by the total number of observations in this subset. The overall model error is taken as the average of the 50 models to the 50 testing subsets (within-sample-error) or to the independent test set using mosquitoes infected in a different location and not used in model training or validation (out-of-sample error). Note that this within-sampling error is more rigorous than other reported out-of-sampling methods which jack-knife data (exclude one sample from the training set each time, and test accuracy on that sample). Sixty-four different parameter combinations were explored for each experiment using a grid search approach in which each of the three smoothing techniques above were considered as binary variables (with 0 implying exclusion and 1 inclusion) with four tuning parameter values τ = 0.05, 0.1, 0.15, 0.2 and three basis sizes k = 25%, 50%, 75% for those models deploying functional representation. The optimal model was then selected by considering the parameter combination producing the minimal overall error in conjunction with those with minimal bias, measured by the absolute value between the false positive and false negative rates. All analyses were carried out in R using the package mlevcm^16^ and assume that diagnostics (microscopy and PCR) are 100% accurate.

## Supplementary Information

Supplementary Figures.

## Data Availability

All data will be placed on an online repository once manuscript is accepted.
