# Supplementary material for: Detection of Plasmodium falciparum in laboratory-reared and naturally infected wild mosquitoes using near-infrared spectroscopy
Source: Sci Rep. 2021 May 13;11:10289. doi: 10.1038/s41598-021-89715-1 (PMC8119679; doi:10.1038/s41598-021-89715-1)
Supplement: Supplementary file 1 — Supplementary Figures. [file 41598_2021_89715_MOESM1_ESM.doc]

Detection of *Plasmodium falciparum* in laboratory-reared and naturally infected wild mosquitoes using near-infrared spectroscopy

**Authors:**

Dari F. Da^1^*, Ruth McCabe^2^, Bernard M. Somé^1^, Pedro M. Esperança^2^, Katarzyna A. Sala^3, 4^, Josua Blight^4^, Andrew M. Blagborough^3^, Floyd Dowell^5^, Serge R. Yerbanga^1^, Thierry Lefèvre^6, 7, 8^, Karine Mouline^6, 7^, Roch K. Dabiré^1,7^, Thomas S. Churcher^2^

**Affiliations:**

^1^ Institut de Recherche en Sciences de la Santé, Direction Régionale, 399 avenue de la liberté, Bobo Dioulasso 01 BP 545 01, Burkina Faso

^2^ MRC Centre for Global Infectious Disease Analysis, Infectious Disease Epidemiology, Imperial College London, London W2 1PG, UK.

^3^ Division of Microbiology and Parasitology, Department of Pathology, Cambridge University, Cambridge CB2 1QP, UK.

^4^ Department of Life Sciences, Imperial College London, Sir Alexander Fleming Building, Exhibition Road, South Kensington, London, UK.

^5^ Stored Product Insect and Engineering Research Unit, United States Department of Agriculture/Agricultural Research Services, Center for Grain and Animal Health Research, Manhattan, Kansas, USA.

^6^ MIVEGEC, Montpellier University, IRD, CNRS, Montpellier, France.

^7^ Laboratoire Mixte International sur les Vecteurs (LAMIVECT), Bobo Dioulasso, Burkina Faso

^8^ Centre de Recherche en Écologie et Évolution de la Santé (CREES), Montpellier, France.

*To whom correspondence should be addressed: [dafrenick@yahoo.fr](mailto:dafrenick@yahoo.fr)

SUPPLEMENTARY MATERIALS

This document contains two supplementary figures:

- Supplementary figure S1
- Supplementary figure S2

**Supplementary figure S1**


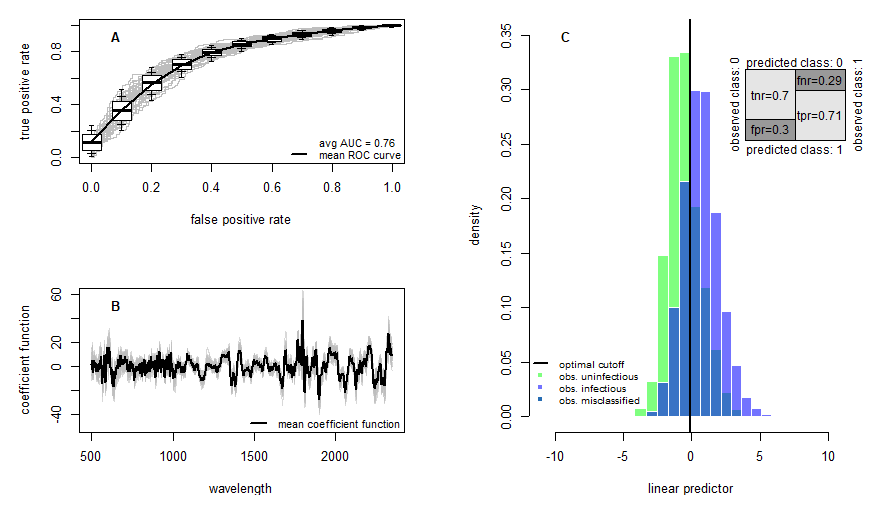


**Fig. S1. The ability of NIRS to predict laboratory-reared mosquitoes infected with wild parasites.** Figure shows differentiation between uninfected mosquitoes and those which have oocyst and/or sporozoites (in comparison to Fig. 1 which shows mosquitoes with or without sporozoites). All models were trained on laboratory reared mosquitoes that were positive for either oocysts and/or sporozoite as opposed that were negative to both life-stages (Table 1). (A) The ROC curve for the best-fit model showing the false positive and true positive rates achievable for different classification probability thresholds whilst the overall performance is given by the AUC. Solid line shows the average ROC curve with boxplots showing the variability for 50 randomizations of the training, validation and testing datasets (with box edges, inner and outer whiskers showing 25th/75th, 15th/85th and 5th/95th percentiles, respectively; and the black line inside the box showing the median/50th-percentile). (B) Coefficient functions for the best fit model for each of the 50 dataset randomizations (grey lines) and the corresponding average (black line). (C) The histogram of the estimated linear predictor for the test mosquitoes, color of the bars indicates the true class, shows the model’s ability to separate the two groups of mosquitoes. The vertical black line indicates the optimum threshold for classifying mosquitoes as infectious or not. The shaded area where the two distributions overlap corresponds to misclassified test observations - false negatives to the left and false positives to the right of the optimal classification threshold. The confusion matrix (inset) shows the different error rates: tnr, true negative rate (specificity); fnr, false negative rate; fpr, false positive rate; and tpr, true positive rate (sensitivity).

**Supplementary figure S2**


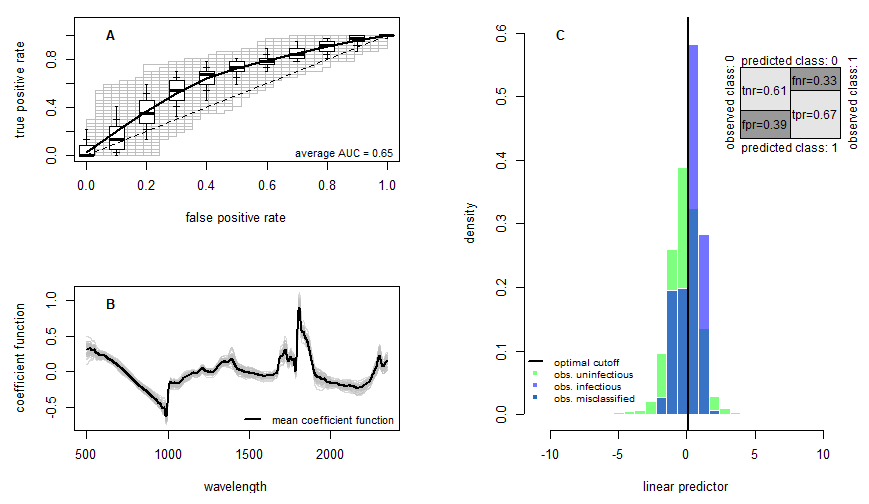


**Fig. S2. The ability of NIRS to predict wild mosquitoes with high sporozoite intensity (>20) using infectious laboratory-reared mosquitoes.** All models were trained on sporozoite positive and sporozoite negative laboratory-reared mosquitoes using all the data presented in Table 1 and applied to the sample of wild mosquitoes with >20 sporozoite gene copy number per ***Anopheles*** (as defined by qPCR) or sporozoite negative mosquitoes (A) The ROC curve for the best-fit model applied to the test mosquitoes showing the false positive and true positive rates achievable for different classification probability thresholds whilst the overall performance is given by the AUC. Solid line shows the average ROC curve with boxplots showing the variability from applying the 50 randomizations of the training model (with box edges, inner and outer whiskers showing 25th/75th, 15th/85th and 5th/95th percentiles; and the black line inside the box showing the median/50th-percentile). (B) Coefficient functions for the best fit model for each of the 50 dataset randomizations (grey lines) and the corresponding average (black line). (C) The histogram of the estimated linear predictor for the test mosquitoes, color of the bars indicates the true class, shows the model’s ability to separate the two groups of mosquitoes. The vertical black line indicates the optimum threshold for classifying mosquitoes as infectious or not. The shaded area where the two distributions overlap corresponds to misclassified test observations - false negatives to the left and false positives to the right of the optimal classification threshold. The confusion matrix (inset) shows the different error rates: tnr, true negative rate (specificity); fnr, false negative rate; fpr, false positive rate; and tpr, true positive rate (sensitivity).
